# Sensitivity Analysis of Different Shapes of a Plastic Optical Fiber-Based Immunosensor for *Escherichia coli*: Simulation and Experimental Results

**DOI:** 10.3390/s17122944

**Published:** 2017-12-19

**Authors:** Domingos M. C. Rodrigues, Rafaela N. Lopes, Marcos A. R. Franco, Marcelo M. Werneck, Regina C. S. B. Allil

**Affiliations:** 1 Federal University of Rio de Janeiro (UFRJ), Electrical Engineering Program, Photonics and Instrumentation Laboratory, Rio de Janeiro 21.941-901, Brazil; domingos@lif.coppe.ufrj.br (D.M.C.R.); rafaela@lif.coppe.ufrj.br (R.N.L.); regina@lif.coppe.ufrj.br (R.C.S.B.A.); 2 Institute of Advanced Studies (IEAv), S. José dos Campos 12.228-001, Brazil; marcos@ieav.cta.br

**Keywords:** biosensor, immunosensor, *E. coli*, fiber optic sensor, POF

## Abstract

Conventional pathogen detection methods require trained personnel, specialized laboratories and can take days to provide a result. Thus, portable biosensors with rapid detection response are vital for the current needs for in-loco quality assays. In this work the authors analyze the characteristics of an immunosensor based on the evanescent field in plastic optical fibers with macro curvature by comparing experimental with simulated results. The work studies different shapes of evanescent-wave based fiber optic sensors, adopting a computational modeling to evaluate the probes with the best sensitivity. The simulation showed that for a U-Shaped sensor, the best results can be achieved with a sensor of 980 µm diameter by 5.0 mm in curvature for refractive index sensing, whereas the meander-shaped sensor with 250 μm in diameter with radius of curvature of 1.5 mm, showed better sensitivity for either bacteria and refractive index (RI) sensing. Then, an immunosensor was developed, firstly to measure refractive index and after that, functionalized to detect *Escherichia coli*. Based on the results with the simulation, we conducted studies with a real sensor for RI measurements and for *Escherichia coli* detection aiming to establish the best diameter and curvature radius in order to obtain an optimized sensor. On comparing the experimental results with predictions made from the modelling, good agreements were obtained. The simulations performed allowed the evaluation of new geometric configurations of biosensors that can be easily constructed and that promise improved sensitivity.

## 1. Introduction

Considering the role of water in human life, it is possible to observe its great potential to spread microorganisms when it is contaminated. Since it is an essential element, water can be the vehicle for the transmission of various types of microorganisms, whether environmentally natural or produced by infected hosts.

Conventional methods for the detection of pathogens require classical techniques that not only involve trained personnel and specialized laboratories, but are also time-consuming. The significant increase in the development and application of biosensor technologies are making it possible to replace these conventional detection techniques, described in the work of Lazkca et al. [1].

In the last decades, several studies concerning optical fiber sensors have arisen and many optical fiber biosensor techniques are still being published [2,3,4,5]. Biosensors that employ antibodies as the biological element of recognition, responsible for their specificity, are called immunosensors. In this work, an immunosensor was prepared with an antibody for the capture of *Escherichia coli*, used as analyte. Previous works developed by our group have shown that these immunosensors allow the identification of *E. coli* suspensions up to 10^4^ colony forming units per milliliter (CFU/mL) [2].

The fundamental physical principle behind the operation of multimode plastic optical fiber (POF) sensors developed in this work is the interaction of the biological sample with the evanescent field along the curved section of the fiber. The bend in the fiber creates a sensitive region to the external environment, causing alterations in the amplitude of the guided light in accordance with the external refractive index (RI), i.e., the analyte to be investigated [6]. Since the optical output power was previously tested in aqueous solutions with known RI, by capturing bacteria that remain bound to the sensor surface, the RI in the sensitive region is altered causing an output power variation. This variation can be related to the calibration slope, enabling the determination of the bacteria concentration around the fiber in that particular sample.

Recent studies have been carried out using this property of U-shaped curved fibers, in which different diameters of conventional POFs with varying radii of curvature are experimentally evaluated [7,8]. Another technique associated with curved fiber characteristics is the development of a taper section, where the sensitive region is tapered for an increased exposure of the fiber core, enhancing the sensitivity. Different taper diameters have already been experimentally tested for macro-curves with different radii, always aiming to increase the sensitivity of the sensor [9].

The use of computational modeling is an indispensable tool for the implementation of improvements in systems, avoiding multiple experimental tests with material expenditure and unnecessary additional costs [10].

In this study, a commercial software (BeamProp^®^—RSOFT Design^®^, Mountain View, CA, USA) based on the Beam Propagation Method (BPM) was used to model the propagation of the optical signal in the biosensor and the consequent power output. The simulations that were performed allowed the evaluation of new geometric configurations of biosensors such as in the work of Fabian et al. [11]. Based on the modeling, new designs of biosensor shapes are suggested in this work, considering the increase of the sensitivity and their technical viability.

In order to validate the results of the simulation, we investigated with a real sensor for RI measurements and for *Escherichia coli* detection. On comparing the experimental results with predictions of the modelling, good agreements were obtained.

## 2. Materials and Methods

### 2.1. Sensing Principle

When the optical fiber is curved, the propagation conditions change because the light rays reach the interface at different angles. Therefore, some modes of propagation escape to the cladding, depending on the external RI. In cladded fibers, some of these modes are guided by the cladding; some other are lost to the surrounding medium. In uncladded fibers the modes that reach the interface with an angle lower than the critical angle, are also lost to the surrounding medium. In both cases, the attenuation of the power output is associated to the curvature radius and the external RI.

Initially, aqueous solutions of sucrose with several refractive indices were produced for testing the sensors. The refractive indices were chosen because they represent refractive indices in a range between pure water (1.33) and a bacterial paste (1.39), the latter representing the maximum refractive index for the sensor completely covered by *E. coli* [12]. Ten measurements were performed between pure water and sucrose solutions, in order to verify the measurement uncertainty.

### 2.2. Sensor Development and Optoelectronic Set-Up

The sensor consists of a poly(methyl methacrylate) (PMMA) core, 980 μm in diameter and a 10 μm cladding, made of an unspecified fluoride polymer. The cladding was removed to expose the PMMA of the core, in which the functionalization was performed as detailed in [12].

The fiber was cut into 10-cm pieces and the cladding was removed by slightly rubbing the region of interest in the fiber, according to the procedure described in [13]. This process was performed at a length of approximately 5.0 mm in the center of the fiber, which represents the region of the sensor to be bent. The POF was then placed in a mold designed to shape fibers in U-shaped form with radii of curvature of 5.0 mm. A hot air jet at about 70 °C is blown over the bent fiber for 25 s. After cooling, the fiber remains curved and both ends were cleaved and polished with emery cloth for better light coupling, as described in detail in [2].

The configuration implemented for the measurement procedure consists of an 830 nm LED connected to one end of the U-Shaped sensor. At the other end, the light is received by a photodiode and amplified by a transimpedance amplifier. An acrylonitrile−butadiene−styrene (ABS) block, made by a 3-D printer, positions the fiber directly in front of the LED and the photodiode. The output signal from the transimpedance amplifier is acquired into an Arduino Microcontroller connected to a computer by an USB cable. The output voltage, proportional to the optical power, is read and shown in the computer display, as detailed in [14].

**Figure 1:**
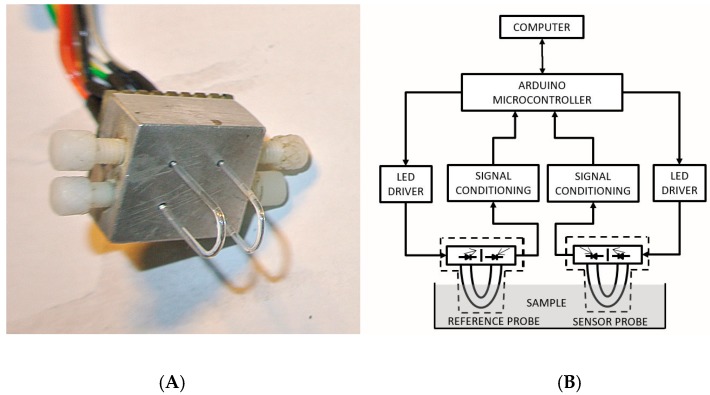
(**A**) Sensor head with two sensors, the reference and the functionalized with antibody. (**B**) Schematic diagram of the optoelectronic setup.

Since the light power output from the sensor is intensity modulated, any variation in the light produced by the LED could interfere with the measurements. Additionally, the probe coupling to the LED or photodiode, temperature oscillations, liquid turbidity, etc. could disturb the output signal. To circumvent these possible variations, we used two parallel probes. One sensor is functionalized for measuring bacteria concentration and the other one, without any functionalization, is used as a reference sensor. The software divides one output signal by the other to produce a referenced signal. Figure 1A shows the sensor head containing two LEDs and two photodiodes with the U-shaped probes attached. Figure 1B shows the optoelectronic setup in a block diagram, built around the Arduino Uno microcontroller board, showing the two similar sensor circuits. The output signals are displayed on the computer, connected to the system by an USB cable.

The tests are made in two steps. In the first step, the sensors are tested under RI variation with their output voltage recorded. Then the sensors are tested in bacteria solutions with their output voltages divided by the output voltage from the reference sensor and recorded by the computer. During bacteria detection, the sensors are immersed in *E. coli* suspension and the measured voltages are recorded every minute for forty minutes. The signal from the biosensor varies according to the bacteria captured by the antibody, while the signal from the reference sensor remains constant.

### 2.3. Funcionalization and Imobilization Protocol of the Immunosensor

The protocol used for binding antibodies on the sensor surface was adapted from [15]. The sensors were cleaned with isopropanol (99%) for 5 min, and then thoroughly washed with sterile distilled water. After washing, the sensors were solubilized in concentrated sulfuric acid at a 1:100 molar for 16 min at room temperature, incubated at 60 °C for 2 h, washed three times with ultrapure water and dried at room temperature. The sensors were then incubated in a solution of 10% hexamethylenediamine prepared with 100 μM borate buffer pH 11.5 for 24 h at 37 °C. After washing three times with ultrapure water, the sensors were dried overnight at 37 °C. To activate amination, the fibers were placed in a 2.5% glutaraldehyde solution prepared in 0.1 M phosphate buffer pH 7.0 for 24 h at 37 °C. After this treatment, the sensors were washed vigorously three times with 0.1 M phosphate buffer, pH 7.0 followed by overnight drying at 37 °C.

Subsequently, the fibers were incubated with 0.05 mg/mL staphylococcal protein A (Sigma-Aldrich, St. Louis, MO, USA), diluted in sodium carbonate (Na_2_CO_3_) buffer pH 9.5 for 1 h at 30 °C. To avoid non-specific binding during processing, binding sites were blocked by treatment with 0.1% bovine serum albumin in 0.85% saline, pH 7.0. Blocking was performed for 1 h at 30 °C under stirring. In sequence, the fibers were washed three times in 0.85% saline, pH 7.0. Finally, the treated fibers were left in contact with 600 μL of *E coli* O55 (0.1 mg/mL) antibody suspension (AbD Serotec, Kidlington, UK) for 4 h at 30 °C, and washed three times in 0.85% saline.

The *E. coli* O55 bacterium used in the preparation of the suspensions was grown on tryptic soy agar (Merck, Darmstadt, Germany) and incubated for 24 h at 37 °C. Subsequently, bacterial suspensions were prepared by adding growth colonies in a tube containing 10 mL of 0.85% saline. The tube was vortexed for homogenization and compared to the turbidity of the McFarland 0.5 scale, equivalent to 10^8^ CFU/mL.

### 2.4. Sample Preparation for Scanning Electron Microscope

The preparation of the sensor with attached bacteria for being viewed under the scanning electron microscope (SEM) followed the Critical Point Drying (CPD) which is an established method of dehydrating biological tissue prior to examination under the vacuum of the SEM. In biological specimens it is important the removal of water because it would cause heat damage to the specimen. Therefore, water has to be replaced by another medium. The most common and convenient transitional medium for critical point drying is carbon dioxide (CO_2_) [16].

The preparation of the samples kept to the following protocol: First, the biological material is fixed on the fiber with 2.5% glutaraldehyde solution in 0.1M sodium phosphate buffer, pH 7.4 for 24 h at 4 °C. Next, the samples undergo a dehydration process being immersed in ethanol in gradual concentrations of 30%, 50%, 70%, 80%, 95% and 100%.

Then, the sensors are immersed in ethanol (100%), and placed in the CPD chamber in which the ethanol is exchanged by liquid CO_2_. During the critical point process, there is a gradual increase in temperature and pressure until the CO_2_ molecules reach the supercritical state. Then, the pressure is reduced until the CO_2_ be converted back to the gas phase. At the end of this processes, the CO_2_ is exhausted to the atmosphere and the sample is dry with the morphological structured preserved [17].

After this process, the samples are metalized with a thin layer of gold deposited by sputtering and ready for the SEM images. The SEM adjustments are 1–30 kV, in high vacuum condition (10^−2^ to 10^−4^ Pa.

### 2.5. Sensor Modelling

In this section the modeling implemented to study and optimize sensitivity of several sensor shapes will be described, based on its diameter and curvature. The modelling was performed by the BeamPROP (RSOFT Design^®^) software, based on Beam Propagation Method that evaluates light propagation along a curved fiber, considering an outside RI. We adopted a two-dimension simulation rather than a 3-D for simplification. For simulation purposes, a spatial transformation technique that allows to represent a curved fiber by means of a straight fiber model was used. A spatial transformation is inserted into the model by varying radially the RI of the material in such a way that the refractive index values decrease in the compressed region and grow in the distended region of the curved fiber. The approach of modelling a curvature by varying the RI is very common and can be seen in many studies of light propagation in waveguides [18].

The preliminary parameters were defined based on the characteristics of the optical fiber structure and the dimensions of the curve. Then, we tested several commercial fiber diameters and curve radius, aiming at achieving an optimized sensor model considering both the fiber and curve dimensions.

**Figure 2:**
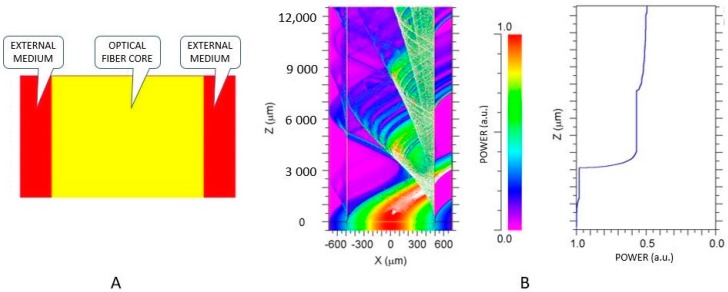
(**A**) Simulation model of a fiber (in yellow) implemented in BeamPROP with a length of approximately 15 mm and radius of curvature of 5.0 mm and surrounding liquids of different refractive indices (red layer). (**B**) Light power distribution inside the fiber. The x-axis of the graph on the right represents the total light power in arbitrary units at each section along the z-axis of the fiber.

Figure 2 shows the model implemented in the BeamPROP for a 980 μm POF (decladed 1-mm diameter ESKA^®^, Mitsubishi, Tokyo, Japan) with a curve of 180° to the left with a radius of curvature of 5.0 mm in radius with a length of about 15 mm. In Figure 2A, it is shown the geometric model representing the fiber core (in yellow) immersed into a liquid with a fixed RI, represented by the red layer at each side of the fiber. A light beam is injected at one extremity of the fiber in a Gaussian distribution. With those initial conditions, the software calculates the light power distribution inside the fiber as shown in Figure 2B. The horizontal axis (x) and vertical axis (z), both in micrometer, plot the light power in every point inside the fiber, represented by the color distribution in which the scale (from zero to 1 a.u.) is shown on the right of the figure. It is possible to observe the formation of interference patterns caused by guided modes interfering with each other inside the fiber. Notice that, as the fiber curves to the left, the light beams go to the right, meaning that the light concentrates at the outside of the curve. In the graph on the right the x-axis represents the light power in arbitrary units at each section along the z-axis. As light propagates along the curved fiber, some guided modes reach the critical angle and refract to the outside of the core. This way, light loses power along the fiber, depending on the RI of the surrounding liquid. Notice that, as the surrounding RI increases, the power output of the fiber decreases because the “V” number, i.e., the number of guided modes in the fiber, is inversely proportional to the difference between the core and cladding refractive indices.

**Figure 3:**
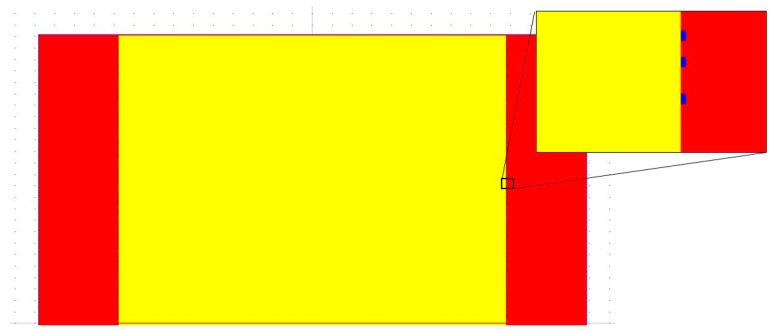
Simulation model of a fiber implemented in BeamPROP in order to simulate a gradual increase of bacteria density (blue rectangles with RI = 1.39) adhered to the fiber surface. The yellow area is the fiber core and the red area the surrounding water.

In Figure 3 we simulated a bacterial layer (blue rectangles with RI = 1.39) around the fiber, simulating a gradual bacteria coverage that occurs during the process of the sensor operation. Each rectangle simulates one bacteria, measuring 2 µm by 1 µm which is the approximate dimensions of *Escherichia coli*.

The process of adhesion of the bacteria occurs in an inhomogeneous way, causing variations in the density of the bacteria layer and a consequent variation in RI. This bacterial layer varies in density (number of bacteria cells per sensor length) and thus varying the average RI from that of pure water (1.33) for zero density, to that of pure bacteria (1.39) for a complete bacteria coverage.

## 3. Results and Discussion

### 3.1. Simulation with RI variations

Simulations were carried out to study the power output behavior of the U-Shaped sensor when both the radius of curvature and the fiber diameter vary. Considering the physical limitations of the optical fiber, its material and all possible curvature radius, the radius of the curvature was simulated between 1.0 mm and 10.0 mm for fiber diameters of 250, 500, 750 and 980 µm. By applying the geometric model shown in Figure 2 we calculated the power output of each combination of fiber diameter/radius of curvature versus the RI of the surrounding liquid.

**Figure 4:**
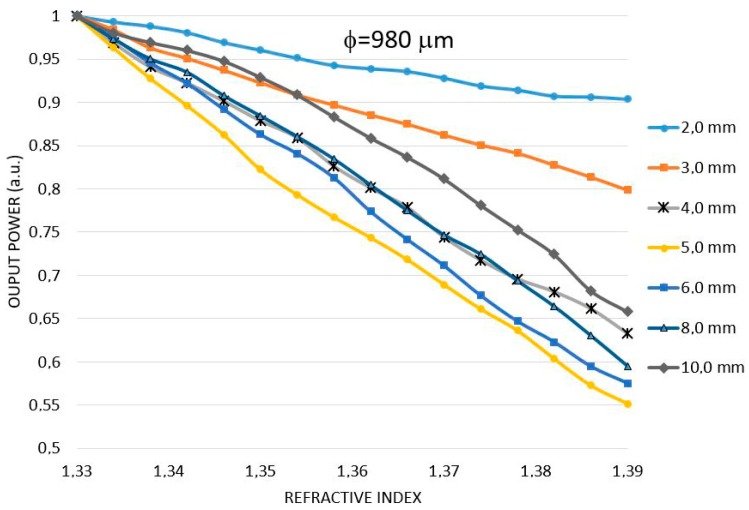
Results of the modelling of U-Shaped sensor for the 980 μm diameter POF. The output power varies as a function of the refractive index for U-shaped sensors with radii of curvature between 2 mm and 10 mm.

Figure 4 shows the simulation of power output vs. RI for a fiber of 980 µm in diameter and different curvature radii. The vertical axis is the normalized power output and the x-axis is the RI of the surrounding media. It is observed that the variation of the power output, i.e., the sensitivity, is not directly proportional to the radius of curvature. Indeed, for curvature radius between 4.0 and 8.0 mm the sensitivity is about the same. For curvature radius of 2.0 and 3.0 and 10.0 the sensitivity is smaller.

The study shown in Figure 4 was repeated for other fiber diameters with all possible curvature radii. Therefore, we simulated sensors with diameters of 250 μm, 500 μm and 750 μm with radii of curvature varying between 1 mm to 10 mm.

Table 1 shows the simulation results for the sensitivity, the variation of optical power (P) to refractive index (n) for all fibers. The underlined numbers indicate the curvature radius for maximum sensitivity for each fiber diameter. The overall maximum sensitivity found was for the fiber of 980 μm with a radius of curvature of 5.0 mm, followed by 250 μm with 1.5 mm and 500 μm with 3 mm.

Notice that, as the fiber diameter decreases, the best radius of curvature also decreases. Therefore, given two fibers with different diameters and bent by the same radius, the one with higher diameter will present the higher sensitivity.

Another interesting conclusion is that, for each fiber diameter, as the radius of curvature decreases, the sensitivity increases up to a maximum and then begins to drop. So, for each fiber diameter there will be an ideal curvature radius for which the sensitivity is maximum.

These simulation results agree with the work of Teng et al. [9] in which the authors studied several fiber diameters and bent curves for RI measurement. For a curve radius of 2 mm, they tested fibers of 600, 400, 250 and 150 μm. The more sensitive sensor was the one with a fiber diameter of 250 μm. Similarly, in another test with a fiber diameter of 250 μm and several bent radii, the more sensitive sensors were the ones with 1 mm and 2 mm. These two results agree with the simulation shown in Table 1.

### 3.2. Experimental Results with RI Variation

For the experimental tests with RI variation we measured the voltage output of the sensors when immersing then into aqueous solutions of sucrose with concentrations of 15%, 25%, 30%, 45% and 52%, previously calibrated with an Abbe refractometer, with RI of 1.35, 1.36, 1.37, 1.38 and 1.39, respectively.

**Figure 5:**
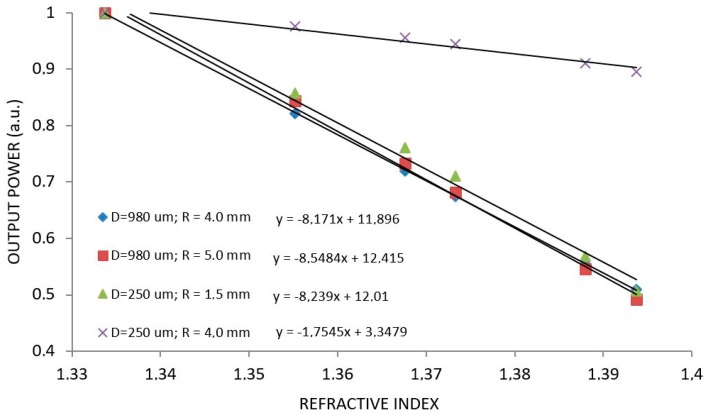
Behavior of U-Shaped sensor with φ = 980 µm for radius of 4.0 mm and 5.0 mm and the sensor with φ = 250 µm for radius of 1.5 mm and 4.0 mm under different RI. The first term of the regression equations is the sensitivity.

Figure 5 shows RI measurements for two sensors that presented best sensitivities: φ = 980 µm with radius of 4.0 and 5.0 mm and sensor of φ = 250 µm for radius of 1.5 and 4.0 mm. This last radius was simulated just for comparison. Regression equations are shown in Figure 5 whose first term are the experimental sensitivity. Notice that all sensors present about the same sensitivity regardless of the diameter or radius. The exception is the 250 µm sensor with 4.0 mm in curvature that performed similarly to a straight fiber, without losing power along the curvature.

### 3.3. Comparison between Simulated and Experimental Results with RI Variation

**Figure 6:**
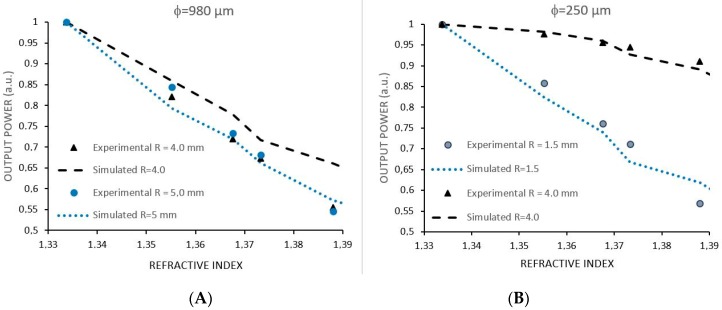
(**A**) Experimental results and simulation of U-Shaped sensors with 980 μm in diameter and radius of 4.0 and 5.0 mm. (**B**) Experimental results and simulation of sensors with 250 μm in diameter and radius of 1.5 and 4.0 mm. The measurement uncertainty is 0.005 RIU (refractive index unit).

In order to compare simulated and experimental results for RI, we superimposed the two respective graphs. Figure 6A shows a comparison between the simulated and experimental results for RI variations for sensor φ = 980 µm for radius R = 4.0 mm and R = 5.0 mm. Figure 6B shows the comparison between the simulated and experimental results using sensor φ = 250 µm for radius R = 1.5 mm and R = 4.0 mm. By making 10 measurements for each point in the graph, we estimated the uncertainty by the worst value of the standard deviation taken for each group of measurements. The measurement uncertainty is 0.00 RIU (refractive index unit).

From Figure 6 it is possible to observe that the experimental and simulated results roughly agree. By fitting correlation curves over each set of data, one can compare the sensitivities as shown in Table 2. Notice that simulated sensitivity is normally smaller than experimental sensitivity since the simulation only takes into account two sides of a simplified sensor, whereas in the experimental tests the interaction of the guided light with the surrounding medium takes place all over the fiber.

### 3.4. Simulation with E. coli

**Figure 7:**
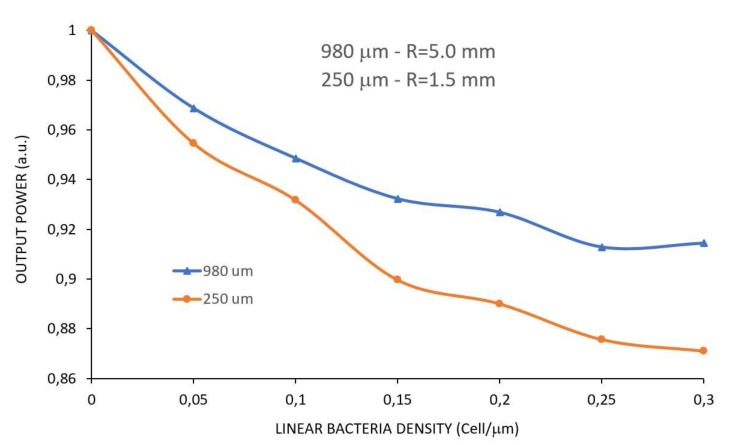
Simulations for φ = 980 µm with R = 5.0 mm and φ = 250 µm with R = 1.5 mm of U-Shaped sensors for a crescent bacteria density along the fiber surface.

As shown in Section 2.4, a layer with variable bacteria density was added along the fiber to simulate the effect of the sensor when capturing bacteria. To be possible to quantify the number of bacteria present in the fiber surface, we defined a variable called linear bacteria density in cells/µm, meaning the number of cells per unity length of the sensor in micrometers. Notice that, since an *Escherichia coli* cell measures about 2 µm in length by 1 µm in diameter, a linear density of about 0.5 cell/µm would completely cover the entire length of the sensor. Therefore, we simulated the sensor based on this information, starting from zero up to a bacteria density of 0.30 cell/µm. Figure 7 shows the simulations for a sensor of φ = 980 µm with radius of R = 5.0 mm and for a sensor of φ = 250 µm with R = 1.5 mm, configurations that showed best sensitivities in the analysis above.

Notice that the 250 µm sensor is more sensitive to bacteria than the 980 µm sensor, differently than when measuring RI, when these configurations presented almost the same sensitivity, as shown in Table 1.

### 3.5. Experimental Results with E. coli

**Figure 8:**
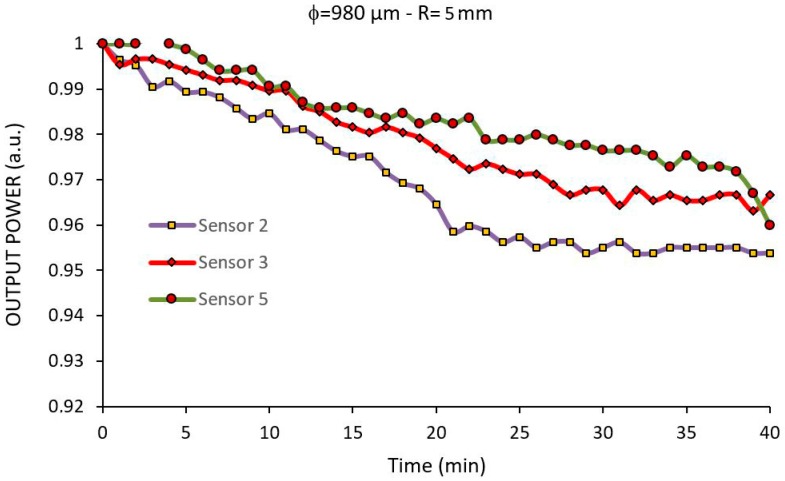
Experimental response of three 980 μm diameter with 5.0 mm curvature radius of U-Shaped sensors for a 10^8^ CFU/mL suspension of E. coli in saline solution.

Five sensors with 980 μm in diameter and 4.0 mm in radius were fabricated, functionalized and immobilized with *E. coli* antibody, as described above. Then, they were, one at a time, immersed in a bacterial suspension of *E. coli* at a concentration of 10^8^ CFU/mL in saline solution for 40 min. Two sensors did not present a stable response due to a bad coupling between the sensor and the LED/photodiode board and their results were discarded. The three other results are show in Figure 8.

We observe that there are different results for the same sensor architecture. This difference can be attributed to the reproducibility of the functionalization of the sensors and the Brownian movement of the bacteria that makes its adhesion to the sensor a random process.

Sensor 3 presented a behavior that represents approximately the average of the other sensors, showing a 3% decrease in the output power after 40 min interaction. Even with a small power drop, previous experiments have shown the possibility of measuring bacterial concentrations as low as 10^4^ CFU/mL with a similar system, but considering that the focus of this work is to study different sensor shapes, only concentrations of 10^8^ CFU/mL were used.

For the visualization of the captured bacteria at the sensor surface, after the experiment the sensors were taken to a scanning electron microscope (SEM).

**Figure 9:**
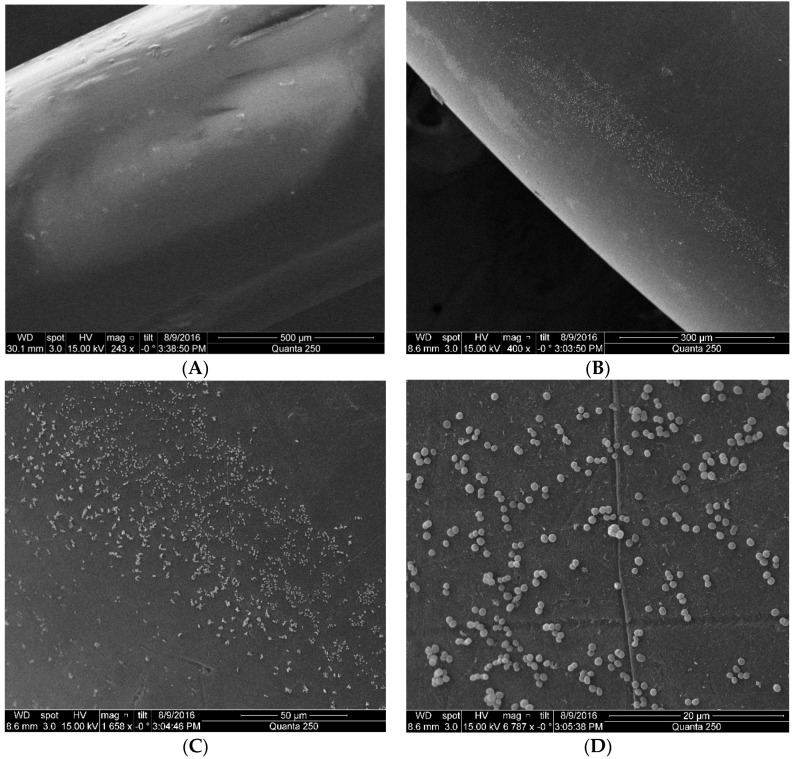
Four pictures with different magnification taken from the sensors under the electron scanning microscope. In (**A**) the picture shows the reference U-Shaped sensor without functionalization under a scale of 500 μm. In (**B**) the U-Shaped sensor functionalized in a suspension of 10^8^ CFU/mL of *Escherichia coli* with a scale of 300 μm. The small dot are the adhered bacteria. (**C**) Bacteria seen at the sensor surface under a scale of 50 μm. (**D**) Under a scale of 20 μm, it is possible to notice individual bacteria distributed along the sensor surface. Each bacterium measures about 2 µm in length by 1 µm in diameter.

Figure 9 shows four pictures with different magnification taken from the sensors. In Figure 9A the picture shows the reference U-Shaped sensor without functionalization under a scale of 500 μm. In Figure 9B the U-Shaped sensor functionalized with a scale of 300 μm in which adhered bacteria is seen as small dots. Figure 9C U-Shaped sensor functionalized where it is possible to see bacteria at the sensor surface under a scale of 50 μm. In Figure 9D with a scale of 20 μm it is possible to notice individual bacteria distributed along the sensor surface.

It is possible to observe in Figure 9D that the bacteria present a round shape instead of the conventional rod shape we are used to see *E. coli*. In order to assure that the cells in our samples were *E. coli* and not any other bacteria species, biochemistry tests were performed to identify the presence of contamination in the growing medium used. All tests showed negative to other bacteria and positive to *E. coli*.

*E. coli* cells are bacilli in a rod shape with rounded ends, with the mean size of 2 µm by 1 µm in diameter. However, they might change their morphology under different environmental conditions, mainly the temperature or growth conditions [19]. The change in shape is associated to the inactivation of some cells and the suppression of proteins involved in cell morphogenesis [20]. The SEM imaging showing rounded cells is justified by the bacterial mutation in its cell division phase, due to the conditions of its growth medium. The slow growth of the elongated part of the bacterium is a response to the protein suppression due to the abrupt thermal variation between bacteria stationary phase in which they are refrigerated at 4 °C and the growth phase in the culture medium at 37 °C.

Notice that the bacteria is sparsely distributed along the sensor surface and that in some parts of the sensor no bacteria is present. This meagerly bacteria concentration explains the small power drop obtained in the experiment shown in Figure 8. Indeed, the average RI at the sensor surface could not be much larger than that of pure water with such a small bacteria concentration.

### 3.6. Comparison between Simulation and Experimental Results with E. coli

In order to compare simulated and experimental results with bacteria, we first noticed that the experimental results are shown as a time response whereas the simulated results are presented against bacteria density. Therefore, to be possible to compare the experimental results shown in Figure 8 with the simulated results shown in Figure 7, we first notice that the minimum power output reached in the experimental test of Sensor 3 (φ = 980 µm-R = 5.0 mm) is about 0.963 a.u. (see Figure 8). We must now locate this value in the simulated result shown in Figure 7 for this sensor. The best way to do so is by fitting a regression curve on the points of Figure 7, yielding:

Y = 1.0047x^2^ − 0.5766x + 0.9969,

where y is the normalized output power and x the bacteria density in cell/µm and the regression coefficient is 0.995.

**Figure 10:**
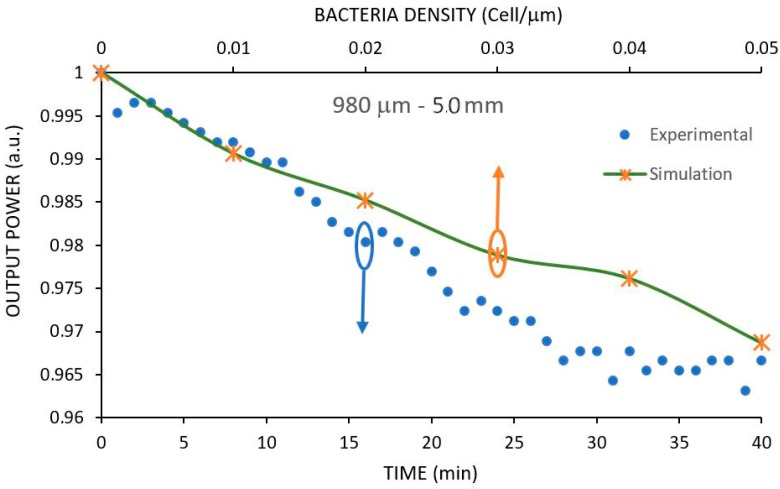
Comparison of the experimental results of the 980 μm-5.0 mm of U-Shaped sensor for *E. coli* with simulated results.

By substituting y = 0.963 into the above equation we get x = 0.0665 cell/µm. This is the cell density in the simulation shown in Figure 7, equivalent to the experimental result shown in Figure 8 after 40 min interaction. Now we superimpose the equivalent section of Figure 7 in Figure 8, yielding the two curves shown in Figure 10.

Notice that the simulation follows the experimental result in the beginning of the interaction but is split away at the end. This difference was expected, as the simulation of a cylindrical fiber was simplified by a two-dimension model that takes into account only the two sides of the model in a longitudinal fashion, whereas in the real world the interaction of the bacteria with the fiber occurs all around it. The other impacting factor in this simulation is the inherit nature of a bacteria cultivate which contains its own uncertainty, leading to different growing patterns.

### 3.7. Simulation of Other Sensors Shapes

Previous studies have shown that the addition of curves in the fiber can increase the sensitivity of the sensor for refractive index measurements, as for instance, the work of Fabian et al. [11]. The authors tested three different sensor shapes for methanol concentrations: U-shaped, coil-shaped and meander-shaped (zigzag) probe. Their study demonstrated that the meander-shaped sensor presented a much higher sensitivity.

**Figure 11:**
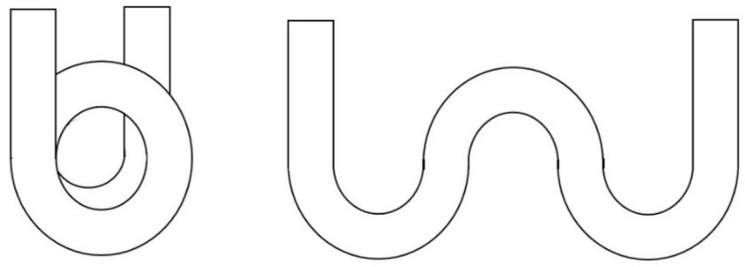
Coil-shaped and a meander-shaped probe models used for simulation.

In order to investigate and evaluate sensitivities of these different forms of sensors, we simulated in our model the coil-shaped and a meander-shaped, of which drawings are shown in Figure 11.

The coil-shaped model contains curves to the same direction, performing one and a half turns, whereas the meander-shaped performs three half turns in opposite directions. In our simulations shown above, the 980 µm-5.0 mm fiber and the 250 μm-1.5 mm fiber presented better sensitivities, and therefore these combinations were used for the simulations of coil-shaped and meander-shaped.

**Figure 12:**
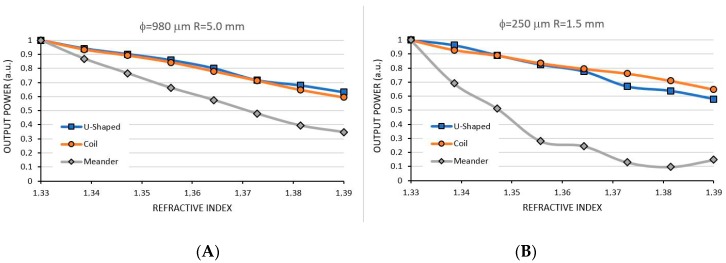
Comparing the simulated sensitivity between U-Shaped, Coil- and Meander-shaped with φ = 980 µm, R = 5.0 mm (**A**) and φ = 250 µm, R = 1.5 mm sensor (**B**) for RI sensing.

Figure 12 shows the simulations for RI sensing for both sensors. In Figure 12A we presented the simulation for the 980 µm-5.0 mm. It is observed that the result for the coil-shaped is similar to the U-shaped sensor, with an output power drop of approximately 40% in the whole RI range. However, the meander-shaped sensor is much more sensitive, with a 90% power drop for the same range. In Figure 12B we show the simulation result for the 250 µm-1.5 mm sensors. Notice that, for the coil-shaped and U-shaped sensors the results are similar to the 980 µm fiber, but for the meander-shaped sensor the simulation shows a much better sensitivity.

**Figure 13:**
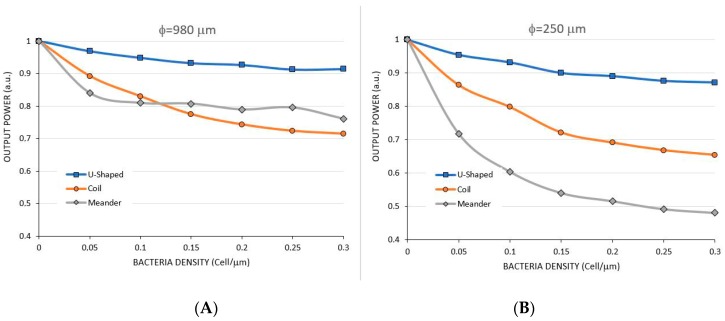
Comparing the sensitivity between U-Shaped, Coil and Meander-shaped with φ = 980 µm, R = 5.0 mm (**A**) and φ = 250 µm, R = 1.5 mm sensor (**B**) for bacteria sensing.

These two sensor diameters were also simulated for bacteria density, as shown in Figure 13. Again, the meander-shaped showed best sensitivity in the range of zero to 0.05 Cell/μm for both fiber diameters. However, notice that, differently from the results in RI sensing, the coil-shaped sensor showed a better sensitivity than the U-shaped sensor.

It is important here to discuss the behavior of the guided light inside each sensor shape to help us to understand the different sensitivities found. When a waveguide curves, the high order modes escape from the core to the surrounding medium. The higher the RI of the surrounding medium, the more modes escape, justifying the loss of power of the sensors as the outside RI increases, either when measuring a variable RI or in a bacteria detection application. Now, comparing the U-shaped sensor and the coil-shaped sensor, the only difference between them is their length. As light propagates inside the fiber and the higher modes are lost, they are not replaced and a longer fiber will not cause any more power losses, justifying the similar results between U-shaped and coil-shaped sensors. However, for bacteria sensing, the longer the fiber the more bacteria it will capture and a longer sensor will lose more light than a shorter sensor for the same bacteria density. This effect is clearly seen in the results above [8].

In the meander shaped fiber, which presented a better sensitivity than all cases above, the light propagation inside the wave guide is slightly different. As the curvature reverses in a zigzag fashion, the waveguide behaves as a mode scrambler. This device is normally used to provide a modal distribution independent of the optical injection source. Therefore, at each curve all lost modes are replaced so that high modes can be lost again at the next curve. In this case, the longer the fiber the higher the power losses with the consequence of an increasing sensitivity. This is true either for the RI measuring or bacteria detection [21]. The drawback of such shape is that it demands a better photodetector sensitivity associated with a higher electronic amplification and noise reduction technique, since the final power output can be as low as 10% of the power input.

## 4. Conclusions

The present work studied different shapes of evanescent-wave based fiber optic sensors. A computational model was used to evaluate the best sensitivity of the sensor probes.

The beam propagation software with a two-dimensional model proved to be a good tool to evaluate evanescent wave based fiber optic sensors. The application of the model simplified and saved time in testing so many parameters that could interfere with the sensitivity, for instance, shapes, curvature radius and dimensions.

The simulation showed that the reduction of the diameter of the fiber in the curve does not necessarily increase the sensitivity, as there is a tradeoff between the fiber diameter and the radius of curvature. The best results were presented by a sensor of 980 μm in diameter with radius of curvature of 5.0 mm, followed by the sensor with diameter 250 µm and 1.5 mm in curvature.

The simulation results for different bending radii and fiber diameters were compatible with the experimental results that were carried out and also agree with data presented by other authors.

The sensitivity of the sensor can be considerably increased by inserting alternating curves in the sensor (meander-shaped sensor). The meander shaped sensor with a fiber of 250 μm increases by 50% the drop in power output for both refractive index measurement and bacteria detection.

The biochemical characteristics of the protocol used to functionalize and bind the antibodies at the sensor surface significantly affect the sensitivity of the immunosensor. One important aspect of the optoelectronic setup is the coupling between the sensors and the LED/photodiode board. The coupling must be stable enough to guarantee steady light transfer to and from the fiber and yet, allow an easy sensor replacement for each new measurement.

Finally, it may be concluded that the low bacteria density shown in the microscopy analyses suggests that the functionalization process of the immunosensor could be improved so that a greater number of bacteria could be captured, allowing a greater sensitivity of the developed sensor. As a future work we are planning to functionalize the meander shaped sensor in order to test it under bacteria concentrations.

## Figures and Tables

**Table 1:** Simulation of the sensitivity of sensors (dP/dn) in the measurement of refractive indices for several diameters and radius of curvature. Bold numbers indicate the best sensitivity for each fiber diameter.

Radius of Curvature (mm)	Fiber Diameter			
	250 μm	500 μm	750 μm	980 μm
1.0	5.67	*	*	*
1.5	**7.30**	*	*	*
2.0	6.61	2.72	1.67	1.70
3.0	4.53	**6.59**	5.24	3.21
4.0	2.37	5.99	7.04	6.12
5.0	1.44	5.41	**7.24**	**7.36**
6.0	0.93	4.44	7.14	7.33
7.0	0.75	3.38	6.80	7.23
8.0	0.64	2.78	5.90	6.66
9.0	0.57	2.45	4.64	6.35
10.0	0.55	2.19	3.57	5.72

(*) Fibers of 500, 750 and 980 μm cannot be bent by 1 mm and 1.5 mm.

**Table 2:** Comparison of simulated and experimental sensitivities.

Sensor (Diameter, Radius of Curvature)	Sensitivity (Simulation)	Sensitivity (Experimental)
980 μm, 5.0 mm	−7.36	−8.55
250 μm, 1.5 mm	−7.30	−8.24
980 μm, 4.0 mm	−6.12	−8.17
250 μm, 4.0 mm	−2.37	−1.74
